# An explicit test of Pleistocene survival in peripheral versus nunatak refugia in two high mountain plant species

**DOI:** 10.1111/mec.15316

**Published:** 2019-12-12

**Authors:** Da Pan, Karl Hülber, Wolfgang Willner, Gerald M. Schneeweiss

**Affiliations:** ^1^ Department of Botany and Biodiversity Research University of Vienna Vienna Austria

**Keywords:** Alps, coalescent simulations, nunataks, peripheral refugia, Pleistocene glaciation

## Abstract

Pleistocene climate fluctuations had profound influence on the biogeographical history of many biota. As large areas in high mountain ranges were covered by glaciers, biota were forced either to peripheral refugia (and possibly beyond to lowland refugia) or to interior refugia (nunataks). However, nunatak survival remains controversial as it relies solely on correlative genetic evidence. Here, we test hypotheses of glacial survival using two high alpine plant species (the insect‐pollinated *Pedicularis asplenifolia* and wind‐pollinated *Carex fuliginosa*) in the European Alps. Employing the iDDC (integrative Distributional, Demographic and Coalescent) approach, which couples species distribution modelling, spatial and temporal demographic simulation and Approximate Bayesian Computation, we explicitly test three hypotheses of glacial survival: (a) peripheral survival only, (b) nunatak survival only and (c) peripheral plus nunatak survival. In *P. asplenifolia* the peripheral plus nunatak survival hypothesis was supported by Bayes factors (BF> 100), whereas in *C. fuliginosa* the peripheral survival only hypothesis, although best supported, could not be unambiguously distinguished from the peripheral plus nunatak survival hypothesis (BF = 5.58). These results are consistent with current habitat preferences (*P. asplenifolia* extends to higher elevations) and the potential for genetic swamping (i.e., replacement of local genotypes via hybridization with immigrating genotypes [expected to be higher in the wind‐pollinated *C. fuliginosa*]). Although the persistence of plants on nunataks during glacial periods has been debated and studied over decades, this is one of the first studies to explicitly test the hypothesis instead of solely using correlative evidence.

## 1 INTRODUCTION

Pleistocene climate fluctuations had profound influence on the biogeographical history of many biota (Hewitt, 1996, 2004). During the glacial periods, large areas in higher latitudes and in high mountain ranges were covered by ice sheets. It is of particular interest to identify where plants and animals occurring in formerly glaciated areas managed to survive during these glacial periods (Gabrielsen, Bachmann, Jakobsen, & Brochmann, 1997; Marr, Allen, & Hebda, 2008; Schönswetter, Stehlik, Holderegger, & Tribsch, 2005; Tremblay & Schoen, 1999; Wachter et al., 2012). In the case of mountain ranges, the focus of the present study, species might have retreated to unglaciated areas at their periphery (peripheral refugia: Holderegger & Thiel‐Egenter, 2009) and possibly beyond into lowlands (lowland refugia: Holderegger & Thiel‐Egenter, 2009), as supported by fossil data (Birks & Willis, 2008) and by molecular data (Comes & Kadereit, 1998; Cosacov, Sérsic, Sosa, Johnson, & Cocucci, 2010; Fulton, Norris, Graham, Semken, & Shapiro, 2013; Marr et al., 2008; Schönswetter et al., 2005; Stehlik, 2000; Tollefsrud, Bachmann, Jakobsen, & Brochmann, 1998) for many plant species. Alternatively, species may have survived within the ice shield on ice‐free mountain peaks, so‐called nunataks (nunatak refugia: Holderegger & Thiel‐Egenter, 2009), as suggested by the nunatak survival hypothesis (Schneeweiss & Schönswetter, 2011; Schönswetter et al., 2005; Stehlik, 2000). Due to a general lack of fossil evidence, nunatak survival is essentially inferred from molecular data only (Lohse, Nicholls, & Stone, 2011; Schönswetter & Schneeweiss, 2019; Stehlik, Blattner, Holderegger, & Bachmann, 2002; Stehlik, Schneller, & Bachmann, 2001; Westergaard et al., 2011). The incidence of nunatak survival may, however, be underestimated, especially in species with high gene flow, because during (re‐)colonization signals of nunatak survival can be genetically swamped by migrants from peripheral refugia (Gabrielsen et al., 1997; Schneeweiss & Schönswetter, 2011; Tollefsrud et al., 1998). The hypotheses of survival in peripheral versus nunatak refugia are, however, not mutually exclusive, and for some species both types of refugia have been inferred (Escobar García et al., 2012; Zhang, Zhu, Zhong, & Zhang, 2018).

**Figure 1:**
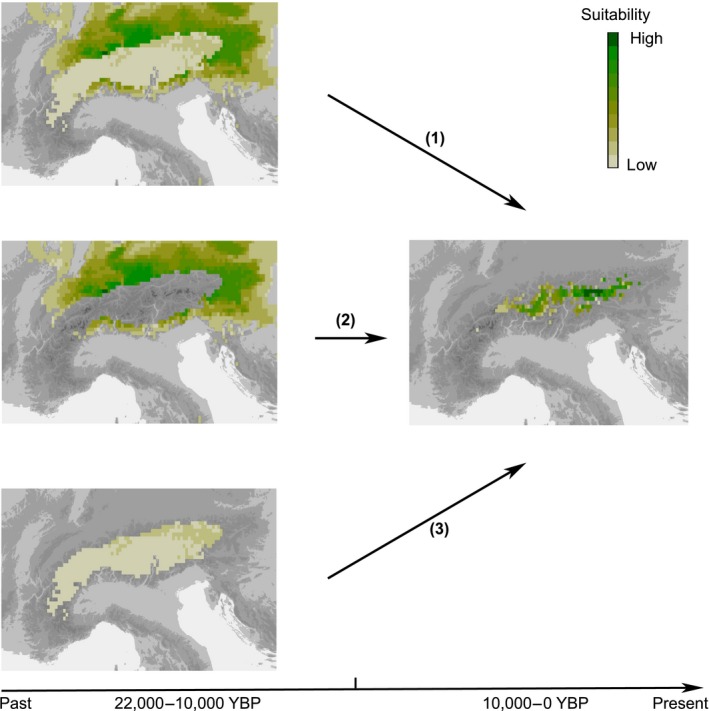
Schematic of the three glacial survival scenarios used in the simulations. Suitability of cells derived from species distribution modelling (SDM) for the Last Glacial Maximum (22,000–10,000 years before present [YBP]) was modified to comply with the different glacial survival scenarios: (1) peripheral plus nunatak survival, (2) peripheral survival only and (3) nunatak survival only; suitabilities for the postglacial (10,000–0 YBP) were taken from the SDM for the present. Grey cells represent unsuitable areas of different altitude [Colour figure can be viewed at http://wileyonlinelibrary.com]

The iDDC (integrative Distributional, Demographic and Coalescent modelling) approach provides a powerful framework allowing different glacial survival scenarios to be explicitly tested (Brown & Knowles, 2012; He, Edwards, & Knowles, 2013; He, Prado, & Knowles, 2017; Papadopoulou & Knowles, 2016). Briefly, using spatially explicit demographic models corresponding to the hypotheses to be tested (which may differ in, for instance, the carrying capacity landscapes or in migration rates), genetic patterns are simulated under the coalescent model. These models, which often are informed by species distribution modelling (SDM), are then evaluated by comparing them to the empirical genetic pattern using an Approximate Bayesian Computation (ABC) framework (for details, see Brown & Knowles, 2012: figure 4). In the context of glacial survival, demographic models differ with respect to whether species are allowed to persist in central glaciated and/or in peripheral unglaciated areas during the glacial period (Figure 1).

**Figure 2:**
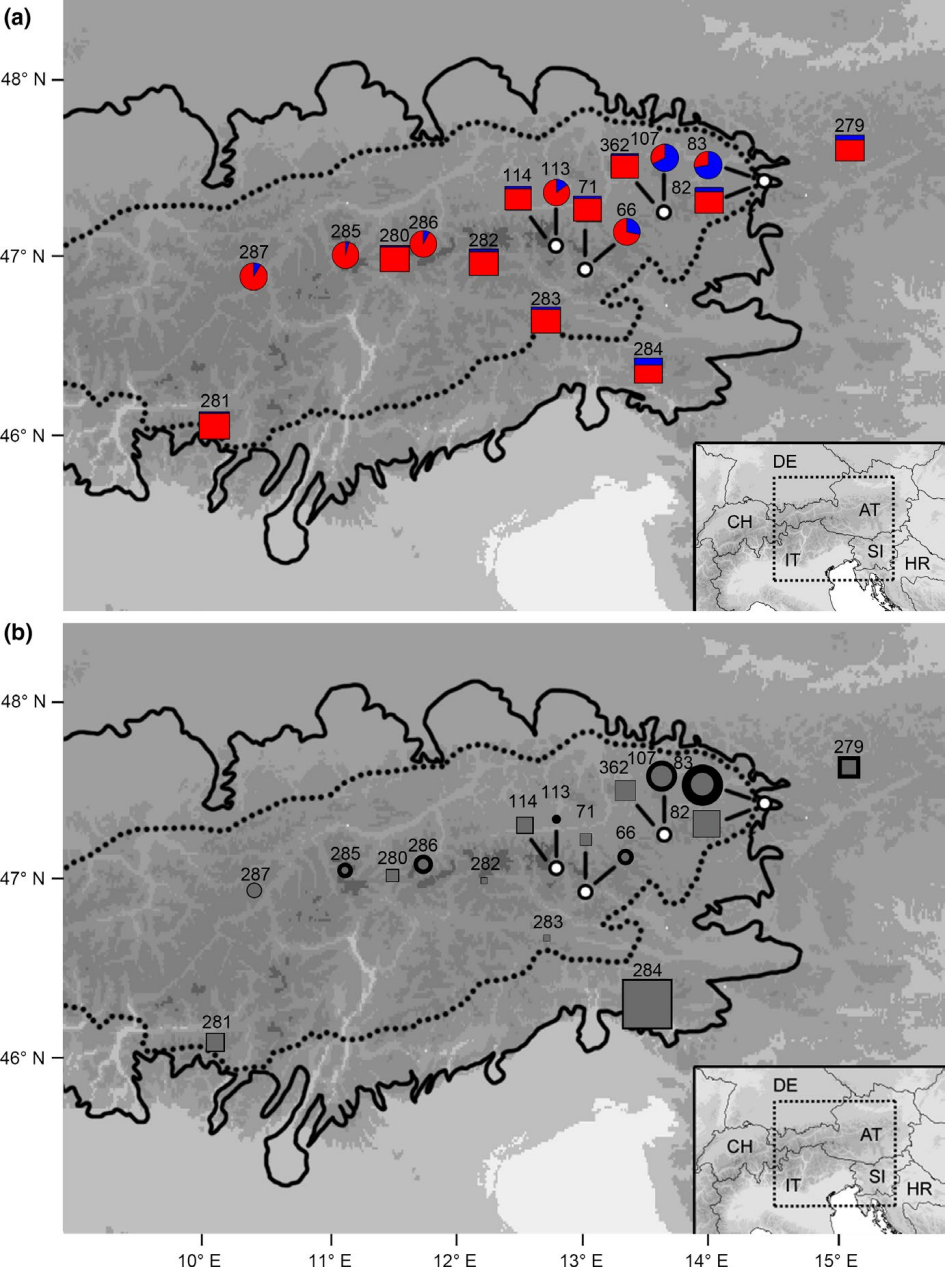
(a) Genetic structure (*K* = 2) and (b) scaled number of private alleles of *Pedicularis asplenifolia* (circles) and *Carex fuliginosa* (squares). In (b), the size of the symbols are in proportion to the scaled number of nearly fixed private alleles (black symbols) and, shown nested therein, the scaled number of fixed private alleles (grey symbols). The dotted line indicates the permanent glacial snow line (i.e., altitude above which snow does not melt in climatically average years) during the Last Glacial Maximum (LGM), and the solid line indicates the maximum extent of the ice‐sheet during the LGM. The right insert shows the position of the study area [Colour figure can be viewed at http://wileyonlinelibrary.com]

In the present study, glacial survival patterns of two plant species, *Pedicularis asplenifolia* and *Carex fuliginosa*, were investigated in the European Alps, a geographical model system to study Pleistocene range shifts (Escobar García et al., 2012; Lohse et al., 2011; Schönswetter et al., 2005). Both species are perennial herbs found exclusively in the alpine and, particularly *P. asplenifolia*, in the subnival belt. As species that can cope with cold harsh environments are likely to be able to survive in extreme habitats such as nunataks (Lohse et al., 2011; Stehlik et al., 2002), they are excellent candidates to test glacial survival hypotheses. In addition, their current distribution ranges encompass both areas situated in formerly glaciated regions, where they may have survived on nunataks, and areas outside the former ice‐sheet, where they may have survived in peripheral refugia (Figure 2). Using RAD‐seq (restriction site associated DNA sequencing) data analysed with the iDDC approach, we here test three glacial survival scenarios identified previously: peripheral survival only, nunatak survival only and peripheral plus nunatak survival (e.g., Escobar García et al., 2012; Schönswetter, Tribsch, Stehlik, & Niklfeld, 2004; Stehlik et al., 2002).

## 2 MATERIALS AND METHODS

### 2.1 Study species

*Pedicularis asplenifolia* (Orobanchaceae) is endemic to the eastern Alps (from eastern Switzerland eastwards through the central Alps in Austria and adjacent parts of northern Italy; Meusel, Jäger, Rauschert, & Weinert, 1978). It is found in the alpine belt (i.e., the belt with closed swards) and the subnival belt (i.e., the belt with open vegetation), where it occurs in open swards and stabilized screes, often on base‐rich schists (Fischer, Oswald, & Adler, 2008). Based on flower morphology, *P. asplenifolia* is considered to be tightly adapted to bumblebee pollination (as in many *Pedicularis* species in the Alps: Heß, 2001), although in practice it appears to be essentially autogameous (Kreisch, 1993).

*Carex fuliginosa* (Cyperaceae) in its narrow circumscription is distributed in the eastern Alps and the Carpathians (Meusel, Jäger, & Weinert, 1965); occurrences in the western Alps, as indicated by Hultén & Fries (1986) for example, are probably erroneous (Schultze‐Motel, 1967–1980; Duhamel, 1994). *C. fuliginosa* is closely related to the circum‐Arctic *C. misandra* (Hultén & Fries, 1986), and both may be merged within one species either recognizing two geographically distinct subspecies or without such intraspecific taxa (Elven, Murray, Razzhivin, & Yurtsev, 2011). *C. fuliginosa* is found in the alpine belt, where it occurs in rocky, usually moist open swards, often on base‐rich schists (Fischer et al., 2008). As with most *Carex* species, *C. fuliginosa* is monoecious and wind‐pollinated (Schultze‐Motel, 1967–1980).

### 2.2 Molecular data generation

Leaf material of 28 *P. asplenifolia* and 46 *C. fuliginosa* individuals was collected from 10 and seven populations, respectively, across the species' entire distributional ranges in the eastern Alps (Table 1; Figure 2). Leaf material was stored in silica gel. DNA extractions were performed following Jang et al. (2013). Single enzyme‐ (*Pst*I) digested RAD libraries (Baird et al., 2008) were constructed using the protocol described by Paun et al. (2015), and sequenced on the Illumina HiSeq2000 platform as single‐end 100‐bp reads at the Vienna Biocenter Core Facilities (https://www.vbcf.ac.at).

**Table 1:** Collection information of the study species

Species	Region	Coordinates^a^	Population number^b^	Number of individuals
*Pedicularis asplenifolia*	A, Rottenmanner und Wölzer Tauern	47°26′/14°25′	83	4
	A, Schladminger Tauern	47°16′/13°38′	107	4
	A, Goldberggruppe	46°57′/13°01′	66	4
	A, Glocknergruppe	47°04′/12°46′	113	4
	A, Zillertaler Alpen	47°05′/11°39′	286	4
	A, Stubaier Alpen	47°02′/11°05′	285	4
	CH, Samnaungruppe	46°54′/10°22′	287	4
*Carex fuliginosa*	A, Hochschwabgruppe	47°36′/15°10′	279	5
	A, Rottenmanner und Wölzer Tauern	47°26′/14°25′	82	5
	A, Schladminger Tauern	47°17′/13°36′	362	4
	I, Julische Alpen	46°22′/13°30′	284	4
	A, Goldberggruppe	46°57′/13°01′	71	5
	A, Glocknergruppe	47°04′/12°46′	114	5
	A, Karnischer Hauptkamm	46°38′/12°42′	283	4
	A, Venedigergruppe	46°59′/12°14′	282	5
	A, Zillertaler Alpen	47°00′/11°33′	280	4
	I, Bergamasker Alpen	46°03′/10°00′	281	5

Abbreviations: A, Austria; CH, Switzerland; I, Italy.

a Latitude/longitude.

b These correspond to collection numbers (“Schneeweiss XY”, where XY is the population number) as used on the vouchers, deposited in the herbarium of the University of Vienna (WU).

Raw reads were demultiplexed by allowing for a single mismatch at the barcodes using illumina2bam (https://github.com/gq1/illumina2bam) and stacks 2.3e (Catchen, Hohenlohe, Bassham, Amores, & Cresko, 2013). Reads with low quality scores (<10) were discarded. Single nucleotide polymorphism (SNP) calling was conducted employing the denovo_map.pl pipeline in stacks (Catchen et al., 2013) with default settings except that the minimum number of identical reads required to build stacks (−m) was set to 5. Loci containing missing data or more than 10 SNPs were filtered out; a cut‐off of 10 SNPs per locus was chosen to avoid a bias towards less variable loci. For final analyses, only one SNP per locus was retained to reduce linkage disequilibrium.

### 2.3 Inferring genetic structure

A Bayesian clustering method implemented in structure 2.3.4 (Pritchard, Stephens, & Donnelly, 2000) was used to identify population structure in the investigated species. Ten independent replicate runs were conducted for each number of populations, *K*, with *K* ranging from 1 to 8. For each run, we ran the admixture model with a burn‐in of 10^5^ generations and sampling from the subsequent 10^6^ generations. The best *K* was selected based on Δ*K* (Evanno, Regnaut, & Goudet, 2005) using structure harvester 0.6.94 (http://taylor0.biology.ucla.edu/structureHarvester/, Earl & vonHoldt, 2012). Structure results were plotted with distruct 1.1 (Rosenberg, 2004).

Relationships among individuals were visualized via a NeighbourNet (Bryant & Moulton, 2004), constructed using HKY85 (Hasegawa, Kishino, & Yano, 1985) distances in splitstree 4.14.2 (Huson & Bryant, 2006), and via a principal component analysis (PCA), conducted in the r package adegenet 2.1.1 (https://CRAN.R-project.org/package=adegenet). The number of fixed (present in all individuals of a population) and the number of nearly fixed (present in at least 75% of all individuals of a population) private alleles for each population were taken from the output of the “populations” function in stacks and corrected by nucleotide diversity as described by Westergaard et al. (2019).

### 2.4 iDDC approach

Species occurrence data were obtained from the GBIF database (https://www.gbif.org/), the project “Mapping the flora of Austria” (H. Niklfeld & L. Ehrendorfer, University of Vienna, unpubl. data) and the European Vegetation Archive (EVA; Chytrý et al., 2016). The accuracy of all georeferenced occurrence data was manually checked, and occurrences outside known distribution ranges were removed, resulting in 126 data points for *P. asplenifolia* and 206 data points for *C. fuliginosa*. Distributions of the two species were modelled for both the present and the last glacial maximum (LGM) period. Nineteen bioclimate variables representing current and past (LGM) climatic conditions were downloaded from the WorldClim database (http://www.worldclim.org/, Hijmans, Cameron, Parra, Jones, & Jarvis, 2005) at a resolution of 2.5 arc minutes. For both studied species, eight bioclimate variables were retained for further analyses (BIO1: annual mean temperature, BIO4: temperature seasonality, BIO8: mean temperature of wettest quarter, BIO9: mean temperature of driest quarter, BIO12: annual precipitation, BIO15: precipitation seasonality, BIO18: precipitation of warmest quarter, BIO19: precipitation of coldest quarter) after removing highly correlated (>0.7) variables. SDMs were calibrated by linking these climatic data to the species occurrence data using the ensemble modelling approach implemented in the package “biomod2” (Thuiller, Georges, Engler, & Breiner, 2016) of r (R Core Team, 2013). Thereby, we selected six modelling techniques: generalized linear model (GLM), generalized boosting model (GBM), generalized additive model (GAM), classification tree analysis (CTA), artificial neural network (ANN) and random forest (RF). To evaluate model quality for each species and modelling technique, the available occurrence data were randomly split into one part to calibrate the models (80%) and the remaining data to evaluate them (20%). To avoid random effects of splitting, we repeated this procedure 10 times. Only models with relative operating characteristic (ROC) values (Swets, 1988) >0.75 were used to subsequently generate ensemble projections of potential species distribution under current climate and under climatic conditions corresponding to the LGM. Ensemble predictions were defined as the means of projected occurrence probabilities of single models. Pseudo‐absence data were randomly generated (i.e., selected from the pool of unoccupied cells) with prevalence equal to 0.5 (i.e., absences have the same weight as presences) with 10 replicates.

Following the approach of Bemmels, Title, Ortego, and Knowles (2016), we up‐scaled the cell sizes of the SDMs from the original 2.5 × 2.5 arc minutes to 5 × 5 arc minutes (i.e., merging four cells resulting in a cell covering ~81 km^2^) using ArcGIS 9 (ESRI). Values for these larger cells were calculated as the mean value from the four smaller cells. Although a finer resolution for a highly heterogeneous landscape such as mountains would be desirable, this would have rendered computation time for the subsequent demographic modelling prohibitively long.

As precise delimitation of refugia is needed for the subsequent modelling, we specifically define interior refugia as areas within the permanent glacial snow line and peripheral refugia as areas outside the permanent glacial snow line, but still within the Alps (Schönswetter et al., 2005; Tribsch & Schönswetter, 2003); because there was only very limited evidence for lowland refugia from species distribution modelling (see Results), these were not considered. Our delimitations of interior and peripheral refugia are more specific than those of Holderegger and Thiel‐Egenter (2009), who explicitly do not consider the permanent glacial snow line to separate peripheral from interior (nunatak) refugia. Peaks protruding above the ice‐sheet were found in essentially all mountain ranges of the eastern Alps, but ice‐free areas were relatively small and isolated (Tribsch & Schönswetter, 2003); hence, studying survival in interior refugia at the level of single nunataks is not possible. Habitat suitabilities at the LGM were modified according to the three main scenarios (Figure 1). Specifically, in the peripheral survival only scenario (Peri), areas of interior refugia (i.e., within the permanent glacial snow line) were considered totally uninhabitable (i.e., suitability was set to zero). The location of the ancestral population (i.e., the geographical starting point for the demographic modelling) was either in the ice‐free eastern part (Peri_East_ scenario) or in the ice‐free southern part (Peri_South_ scenario) of the Alps (corresponding to refugium IV and refugium III, respectively, of Schönswetter et al., 2005). In the nunatak survival only scenario (Nun), suitabilities in areas of interior refugia were reduced by 85% (as in Massatti & Knowles, 2016) and those in peripheral areas (i.e., outside the permanent glacial snow line) were set to zero; an ancestral population in the central glaciated area of the Alps was used. In the peripheral plus nunatak survival scenario (Peri + Nun), the suitabilities of cells of interior refugia was decreased by 85%, while those of peripheral refugia were left unchanged. The single ancestral population was located either in the ice‐free eastern part (Peri_East_ + Nun scenario), the ice‐free southern part (Peri_South_ + Nun scenario) or the central glaciated part of the Alps (Peri + Nun_Central_ scenario); using two ancestral populations was computationally not feasible with the available resources.

**Figure 3:**
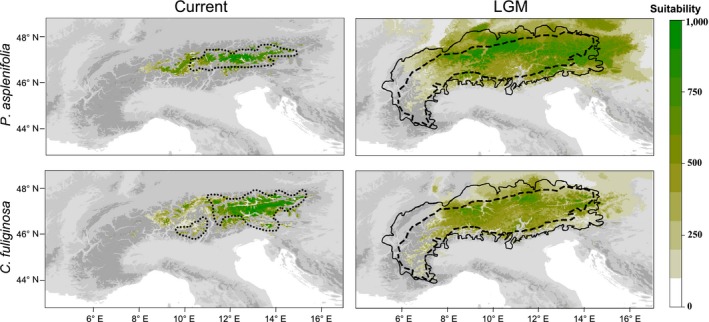
Projected suitabilities of *Pedicularis asplenifolia* and of *Carex fuliginosa* under current climate conditions (left panel) and under climate conditions at the Last Glacial Maximum (LGM; right panel). In the left panel, the dotted lines indicate the current distribution ranges of the two species; in the right panel, the dashed lines indicate the permanent glacial snow line during the LGM and the solid lines indicate the maximum extent of the ice‐sheet during the LGM [Colour figure can be viewed at http://wileyonlinelibrary.com]

For each scenario, 10^6^ demographic simulations were performed in splatche 3.0 (Currat, Arenas, Quilodran, Excoffier, & Ray, 2019). The generation times of both species were set to 40 years (resulting in 550 generations for the considered time period from 22,000 years before the present until the present), following a rescaling approach (the actual generation times are probably considerably shorter) similar to the one used by Massatti and Knowles (2016). The main reason for rescaling generation time is to make simulations computationally feasible, because increasing generation time results in fewer generations being simulated. The actual generation time will not affect model evaluations, but any interpretation of the estimated parameters would have to take the rescaling and possible differences in generation times between the two species into account (Massatti & Knowles, 2016). Demographic parameters, including migration rate *m* (the per‐generation probability of an individual moving out of a deme [i.e., grid cell] into neighbouring demes), maximum carrying capacity *k*, and population size of ancestral population *Nanc*, were drawn from uniform priors through abctoolbox 2.0 Beta (https://bitbucket.org/phaentu/abctoolbox-public/, Wegmann, Leuenberger, Neuenschwander, & Excoffier, 2010); these priors were sufficiently broad to prevent posteriors being unduly affected by narrow prior bounds. Specifically, the priors were *m* ~ U(0.001, 0.1), *k* ~ U(5 × 10^2^, 2 × 10^4^), *Nanc* ~ U(2 × 10^3^, 5 × 10^6^) for an ancestral population located in a peripheral area and *Nanc* ~ U(5 × 10^2^, 5 × 10^4^) for an ancestral population located in the glaciated central Alps, where populations are expected to have been smaller. The prior on the migration rate was set following the considerations of Bemmels et al. (2016). Specifically, the minimum value of the migration rate was chosen to allow the (climatically) suitable landscape to be fully colonized, and the maximum value was chosen to avoid colonization being too rapid, as this would eliminate any differences between models. However, the upper bound of the migration rate was set higher than in Bemmels et al. (2016) to compensate for scaled generation time (larger generation times result in fewer generations being simulated and thus may require higher migration rates). Cells with suitability <10% of the maximum were treated as totally unsuitable (i.e., their suitabilities were set to zero) to remove nonzero, although small, suitabilities mostly outside the mountain ranges (Figure 3). Subsequently, habitat suitabilities derived from the SDMs larger than zero were classified into 10 categories in increments of 10% of the maximum suitability found in the particular species using a modified python script from x‐origin (He et al., 2017). The carrying capacity of each cell was scaled according to its suitability. From generation 1 (22,000 years before the present) to 300 (10,000 years before the present), corresponding to the glacial period, demographic modelling used the modified SDM predictions for the LGM, as described above, whereas for generations 301–550 (present time) the modelling used the SDM predictions based on the current climate.

Genetic data sets matching the dimensions of the empirical data set (i.e., having the same number of samples and SNPs) were generated on each of the 10^6^ demographic simulations (i.e., using the stored demographic and migration histories of each grid cell at each generation) using splatche 3.0 (Currat et al., 2019). To this end, SNPs were considered unlinked and the minimum frequency of SNPs was set to zero; simulation of SNP data in splatche does not require a mutation rate to be specified (see manual available from http://www.splatche.com/splatche3). For both simulated and empirical data sets, summary statistics were calculated in arlequin 3.5 (Excoffier & Lischer, 2010), including mean number of alleles over loci for each population, mean number of alleles over loci and population, mean heterozygosity over loci for each population, mean heterozygosity over loci and population, mean total heterozygosity and pairwise population *F*_ST_. A total of 38 and 68 summary statistics were computed for *P. asplenifolia* and *C. fuliginosa*, respectively. These numbers differ because of the different number of populations analysed for the two species.

To identify the best supported scenario we employed ABC with abctoolbox 2.0 Beta (Wegmann et al., 2010). Instead of using all computed summary statistics directly, we converted summary statistics to partial least squares (PLS) components using the r package “pls” (Mevik & Wehrens, 2007) with Box–Cox treatment (Box & Cox, 1964). The number of PLS components to be used was determined based on the root mean squared error (RMSE) plots. For each scenario, 5,000 (0.5%) simulated genetic data sets that are closest to the empirical data set were retained for parameter estimation and model selection. A post‐sampling regression adjustment was applied using GLMs (Leuenberger & Wegmann, 2010). Marginal densities were used to evaluate models. For validation, *p*‐values were calculated to check if the models are able to generate the empirical data (Wegmann et al., 2010). Additionally, we checked whether parameter estimations are unbiased using 1,000 pseudo‐observations; a uniform distribution of posterior quantiles is expected if estimation of the parameter is unbiased (Wegmann et al., 2010).

## 3 RESULTS

### 3.1 RAD sequencing

After demultiplexing and quality filtering, more than 590,000 reads were obtained per individual (available under accession numbers SAMN13021464–SAMN13021537). After filtering of loci and retaining only one SNP per locus, the final data sets contained 2,185 SNPs for *Pedicularis asplenifolia* and 2,486 SNPs for *Carex fuliginosa* (Pan, Hülber, Willner, & Schneeweiss, 2019; available on Dryad at https://doi.org/10.5061/dryad.rg0134r).

### 3.2 Genetic structure

For both *P. asplenifolia* and *C. fuliginosa*, Δ*K* suggested *K* = 2 as the most likely number of groups (Figure S1). In *P. asplenifolia* these groups showed geographical structure and were longitudinally separated, whereas in *C. fuliginosa*, these groups showed no biologically easily interpretable structure, as the proportion of membership to the minor cluster in any individual was less than 0.4 (Figure 2a; Figure S2).

In the Neighbor‐Net of *P. asplenifolia*, three groups could be distinguished, corresponding to each of the two easternmost populations (pop. 83 and pop. 107, belonging to the same genetic cluster: Figure 2a) and to the remaining populations (Figure S3). The same three groups were identified in the PCA, where pops. 83 and 107 were separated from the other populations along the first axis (explaining 25.2% of the variation) and pops. Eighty‐three and 107 were separated from each other along the second axis (explaining 13.2% of the variation; Figure S4). In the Neighbor‐Net of *C. fuliginosa*, populations were clearly separated from each other, yet there was no major split suggesting any grouping among populations (Figure S3). In the PCA, pop. 284, supported by a long split in the Neighbor‐Net (Figure S3), was separated from the remaining populations along the first axis (explaining 14.3% of the variation), and the remaining populations were separated, mostly longitudinally, along the second axis (explaining 9.8% of the variation; Figure S4).

The numbers of (nearly) fixed private alleles were high in (nearly) peripheral populations both in *P. asplenifolia* (pops. 83 and 107) and in *C. fuliginosa* (especially pop. 284, less so in pops. 82, 279 and 281; Figure 2b, Table S1). The numbers of (nearly) fixed private alleles were constantly lower in each of the interior populations, but (except for the number of private alleles in pop. 113 of *P. asplenifolia*) were always larger than zero (Table S1) occasionally approaching levels of peripheral populations (e.g., interior pop. 114 vs. peripheral pop. 281 of *C. fuliginosa*; Figure 2b, Table S1).

### 3.3 Model evaluation

Based on current climate data, SDM predicted suitable areas for *P. asplenifolia* and *C. fuliginosa* that were mostly congruent with their current distribution ranges (Figure 3). According to the projections at LGM conditions, major parts of the Alps as well as peripheral areas (mostly adjacent midelevation mountain ranges) were suitable for *P. asplenifolia* and, to a lesser extent, also for *C. fuliginosa* (Figure 3).

Observed heterozygosity, *H*_obs_, and expected heterozygosity, *H*_exp_, ranged in *P. asplenifolia* from 0.1536 to 0.2368 and from 0.0958 to 0.1708, respectively; in *C. fuliginosa*, they ranged from 0.1372 to 0.1624 and from 0.0814 to 0.1196, respectively (Table S1). The mean heterozygosity over loci and population, *H*, ranged from 0.1094 to 0.1951 in *P. asplenifolia* and from 0.0930 to 0.1328 in *C. fuliginosa* (Table S1); accordingly, both the mean heterozygosity over loci and population, *H*_mean_, as well as the total heterozygosity, *H*_total_, were higher in *P. asplenifolia* than in *C. fuliginosa* (*H*_mean_: 0.1455 vs. 0.1112, respectively; *H*_total_: 0.1729 vs. 0.1413, respectively). The mean number of alleles over loci and population, *K*, ranged from 1.2485 to 1.4622 in *P. asplenifolia* and from 1.2071 to 1.3660 in *C. fuliginosa* (Table S1); accordingly, the mean number of alleles over loci and population, *K*_mean_, was higher in *P. asplenifolia* than in *C. fuliginosa* (1.3447 vs. 1.2885, respectively). Pairwise *F*_ST_ values ranged from 0.008 to 0.332 in *P. asplenifolia* and from 0.087 to 0.422 in *C. fuliginosa* (Table S2).

Based on the RMSE plots (Figure S5), three to five PLS components were retained for calculating the distance between simulated and empirical data sets. In *P. asplenifolia*, the peripheral plus nunatak survival scenario with the ancestral population located in the eastern Alps (Peri_East_ + Nun) best explained the empirical genetic pattern (Table 2). All remaining scenarios were clearly rejected (Bayes factor [BF] support for the best model in all cases >100). In accordance, the Peri_East_ + Nun scenario better reproduced the empirical data (*p* = .736) compared to all alternative scenarios (*p* ≤ .001). In *C. fuliginosa*, the best supported model was the peripheral survival only scenario with the ancestral population located in the eastern Alps (Peri_East_; Table 2), followed by the peripheral plus nunatak survival scenario with the ancestral population located in the eastern Alps (Peri_East_ + Nun; BF support for the best model = 5.58). For these models, *p*‐values were .993 and .992, respectively (Table 2). The remaining scenarios were clearly rejected (BF support for the best model >100) and had *p*‐values ≤ .001. In both species, prior distributions of parameter estimates in the best supported models were distinct from the posterior distributions (Figure S6), indicating that the data have power to estimate parameters. Parameter estimates were not unbiased, as posterior quantiles of all parameters departed from a uniform distribution (Kolmogorov–Smirnov test, Figure S7).

**Table 2:** Comparison of Pleistocene survival scenarios of the study species

Species	Model^a^	Marginal density	Bayes factor^b^	*p*
*Pedicularis asplenifolia*	Nun	1.28 × 10^−76^	>100	<.001
	Peri_East_ + Nun	2.57 × 10^−5^	–	.736
	Peri_South_ + Nun	<1.00 × 10^−100^	>100	<.001
	Peri + Nun_Central_	<1.00 × 10^−100^	>100	<.001
	Peri_East_	2.48 × 10^−13^	>100	<.001
	Peri_South_	<1.00 × 10^−100^	>100	<.001
*Carex fuliginosa*	Nun	8.05 × 10^−97^	>100	<.001
	Peri_East_ + Nun	1.51 × 10^−4^	5.58	.992
	Peri_South_ + Nun	<1.00 × 10^−100^	>100	<.001
	Peri + Nun_Central_	2.00 × 10^−96^	>100	<.001
	Peri_East_	8.43 × 10^−4^	–	.993
	Peri_South_	<1.00 × 10^−100^	>100	<.001

a Nun, nunatak survival in interior refugia, the index indicating (where necessary) the location of the ancestral population (Central, central Alps); Peri, peripheral survival in peripheral refugia, the index indicating (where necessary) the location of the ancestral population (East, eastern Alps; South, southern Alps).

b The ratio between marginal densities of the best model (i.e., the one with the highest marginal density) and of the alternative model: the higher the value, the higher the support for the best model.

## 4 DISCUSSION

Concerning the debate as to whether nunatak survival does matter, the answer may be species‐specific rather than universal (Gabrielsen et al., 1997; Tollefsrud et al., 1998; Wachter et al., 2016; Westergaard et al., 2011). Species traits affecting, for instance, dispersal capabilities shape current genetic patterns both through glacial survival per se (via, for instance, genetic bottlenecks; Schönswetter, Paun, Tribsch, & Niklfeld, 2003; Wachter et al., 2012) and through post‐glacial recolonization (via, for instance, gene flow or long‐distance dispersal; Paun, Schönswetter, Winkler, IntraBioDiv Consortium, & Tribsch, 2008; Schönswetter, Tribsch, Barfuss, & Niklfeld, 2002). As shown in this study, although *Pedicularis asplenifolia* and *Carex fuliginosa* have similar habitat preferences and current distribution ranges, unambiguous evidence for nunatak survival was only found in *P. asplenifolia*.

In *P. asplenifolia*, both peripheral and nunatak areas appear to have acted as refugia during the LGM. This is evident from the support for the peripheral plus nunatak survival scenario with the ancestral population located in the eastern Alps (Peri_East_ + Nun; Table 2), an area that acted as a glacial refugium also for other alpine plants (Schönswetter et al., 2005). Peripheral and nunatak survival were shown to jointly contribute to current genetic patterns in some high alpine plants (Escobar García et al., 2012; Schönswetter & Schneeweiss, 2019), and this appears also to be the case in *P. asplenifolia*.

In contrast, in *C. fuliginosa*, only peripheral areas in the easternmost Alps could be unambiguously confirmed as refugia, although based on BFs a nunatak survival cannot be ruled out (Table 2). A lack of nunatak survival in *C. fuliginosa* would agree with, compared with *P. asplenifolia*, a lower tolerance against harsh climate conditions expected to have occurred at Pleistocene nunataks. Such a lower tolerance is suggested by the current altitudinal distributions, as *C. fuliginosa* is restricted to the alpine zone whereas *P. asplenifolia* frequently extends into the subnival zone. Alternatively, however, the ambiguity with respect to nunatak survival might be because traces of nunatak survival may have been genetically swamped (Gabrielsen et al., 1997; Tollefsrud et al., 1998) after (re‐)colonization following deglaciation and subsequent gene flow between immigrants from peripheral refugia and in situ inhabitants in *C. fuliginosa*. The potential for genetic swamping due to gene flow is expected to be higher in wind‐pollinated species (Govindaraju, 1988) such as *C. fuliginosa* than in insect‐pollinated species such as *P. asplenifolia*. Genetic homogenization may also be responsible for the lack of geographical structure in the genetic data despite the presence of geographically distant populations in *C. fuliginosa* (Figure 2; Figures S2–S4). Wind‐pollination has been shown to mediate post‐glacial gene flow among refugia (Liepelt, Bialozyt, & Ziegenhagen, 2002). For taxa prone to genetic swamping, neither a correlative genetic approach nor a modelling approach as used here may allow nunatak survival to be detected, even if based on genomic data such as RAD‐seq data. In those cases, valuable information may be obtained by using mostly uniparentally inherited markers not prone to homogenization, as is the case for plastid or mitochondrial sequences (Schönswetter & Schneeweiss, 2019).

We acknowledge that our sampling is less intensive (four or five individuals per population) compared to other studies (e.g. Bemmels et al., 2016; Massatti & Knowles, 2016; Westergaard et al., 2019). This may, however, be compensated for by a larger number of SNPs (2,185 SNPs for *P. asplenifolia* and 2,486 SNPs for *C. fuliginosa*) when only retaining loci without any missing data as done here. Comfortingly, analyses of an earlier data set with fewer individuals (two individuals per population), but more SNPs (5,504 SNPs for *P. asplenifolia* and 4,976 SNPs for *C. fuliginosa*) resulted in the same ranks for the glacial survival scenarios as identified here, yet with reduced decisiveness (i.e., lower BFs; Table S3). This indicates that our inferences are robust with respect to sampling intensity.

As with any ABC approach, model validation is essential, because ABC will always produce posterior distributions independent of model quality (Bertorelle, Benazzo, & Mona, 2010; Wegmann et al., 2010). In our case, compared to alternative models, the most supported model had higher probability of generating data similar to the empirical one (high *p*‐values) than the alternative models, indicating that the model evaluation results are robust. The posterior quantiles from pseudo‐observations of all estimated parameters showed departure from a uniform distribution (Figure S7), suggesting that they are estimated inaccurately and that their biological interpretation should be avoided (Wegmann et al., 2010). However, in this study, we were not interested in the specific parameter values, as our primary objective was to distinguish alternative glacial survival scenarios.

Utilizing several summary statistics simultaneously, although not free from potential problems (e.g., the curse of dimensionality; Beaumont, 2010), is advantageous, as single summary statistics may not suffice to distinguish different evolutionary hypotheses (Hickerson, Dolman, & Moritz, 2006; Lin, Li, Schlötterer, & Futschik, 2011). This appears also to be the case for private alleles, which are expected to be high in populations that have experienced genetic drift in isolated refugia (Hewitt, 2004; Westergaard et al., 2019) and thus are good indicators for the position of refugia. In line with a hypothesis of peripheral refugia, the corrected numbers of (nearly) fixed private alleles were high in peripheral populations of both species (Figure 2b; Table S1). Evidence for or against nunatak survival from the distribution of (nearly) fixed private alleles was, however, ambiguous, because the corrected numbers of (nearly) fixed private alleles were nearly always above 0 (Table S1) and occasionally approached levels of peripheral populations. Only *after* taking the results from the iDDC approach into account, the elevated numbers of (nearly) fixed private alleles in pop. 286 of *P. asplenifolia* may be interpreted as indicating an interior refugium in the central Alps (as suggested previously: Escobar García et al., 2012; Schönswetter & Schneeweiss, 2019).

We acknowledge that testing more refined scenarios with respect to, for instance, geographical resolution of nunataks or model parameterization would be desirable, but there will be data‐imposed limits. Regardless, testing simple models does not compromise the biological insights from our study, which is that nunatak survival during periods of glaciation, at least potentially, contributed to the biogeography and evolution of alpine plant species.

## 5 CONCLUSION

In phylogeographical studies, multiple demographic histories may lead to similar genetic patterns. For example, high genetic diversity may be the outcome of either secondary contact or of temporally stable populations (Nettel, Dodd, Afzal‐Rafii, & Tovilla‐Hernández, 2008; Ursenbacher et al., 2008), or both geographical isolation and founder events might generate high genetic differentiation between populations (Gugerli et al., 2001; Schönswetter, Popp, & Brochmann, 2006). Using model‐based approaches as applied here allows the genetic pattern to be explicitly linked to the phylogeographical history of species (He et al., 2013; Massatti & Knowles, 2016). Thus, we could unambiguously demonstrate nunatak survival within the heavily glaciated central Alps in *Pedicularis asplenifolia*. Although the persistence of plants on nunataks during glacial periods has been debated and studied over decades (Gabrielsen et al., 1997; Schneeweiss & Schönswetter, 2011; Tollefsrud et al., 1998; Westergaard et al., 2011), this is one of the few studies (e.g., Westergaard et al., 2019) to explicitly test the hypothesis instead of solely using correlative evidence.

## AUTHOR CONTRIBUTIONS

D.P. and G.S. conceived the study; D.P. and G.S. collected specimens; W.W. provided distribution data; K.H. advised on SDM methods; D.P. conducted laboratory work and data analyses; D.P., K.H., W.W. and G.S. wrote the manuscript.

## Data Availability

Illumina sequence reads for *Pedicularis asplenifolia* and *Carex fuliginosa* are available under BioProject PRJNA577188, and genetic data (SNP data) are available at Dryad (https://doi.org/10.5061/dryad.rg0134r).
