# Supplementary material for: An explicit test of Pleistocene survival in peripheral versus nunatak refugia in two high mountain plant species
Source: Mol Ecol. 2019 Dec 12;29(1):172–83. doi: 10.1111/mec.15316 (PMC7003806; doi:10.1111/mec.15316)

MOLECULAR ECOLOGY

Supplemental Information for:

An explicit test of Pleistocene survival in peripheral versus nunatak refugia in two high mountain plant species

Da Pan, Karl Hülber, Wolfgang Willner, Gerald M. Schneeweiss

Table of Contents:

Table S1: Summary statistics for each population 2

Table S2: Pairwise F_{ST} values 3

Table S3: Comparison of Pleistocene survival scenarios using two individuals per population 4

Figure S1: Plots of Delta *K* and of mean $Log_e(X|K)$ for each *K* value 5

Figure S2: Genetic structure (*K*=2) estimated using STRUCTURE 6

Figure S3: Neighbor-Net 7

Figure S4: Scatter-plots of the first two components of a PCA 8

Figure S5: Change in Root Mean Square Error (RMSE) with increasing number of Partial Least Squares (PLS) components for the most supported model 9

Figure S6: Prior and posterior distributions of parameter estimates 10

Figure S7: Distribution of posterior quantiles of parameters 11

MOLECULAR ECOLOGY

Table S1 Summary statistics for each population of *Pedicularis asplenifolia* and *Carex fuliginosa* based on SNP data.

	pop[1]	Summary statistics[2]					
		H_{obs}	H_{exp}	H	π	K	A_p
P. asplenifolia	83	0.2368	0.1708	0.1951	0.1952	1.4622	52.4 / 15.6
	107	0.1965	0.1366	0.1561	0.1562	1.3725	29.5 / 13.7
	66	0.2223	0.1484	0.1696	0.1696	1.3986	8.2 / 2.1
	113	0.1833	0.1220	0.1394	0.1395	1.3308	2.6 / 0
	286	0.1536	0.0958	0.1094	0.1095	1.2485	11.6 / 3.3
	285	0.1658	0.1038	0.1186	0.1186	1.2901	7.6 / 1.5
	287	0.1844	0.1138	0.1301	0.1301	1.3103	6.8 / 5.5
C. fuliginosa	279	0.1577	0.1196	0.1328	0.1329	1.3660	15.7 / 6.3
	82	0.1550	0.1030	0.1145	0.1145	1.3105	22.1 / 20.9
	362	0.1372	0.0814	0.0930	0.0930	1.2071	12.3 / 12.3
	284	0.1432	0.0862	0.0984	0.0985	1.2506	75.9 / 66.0
	71	0.1624	0.1044	0.1160	0.1160	1.3181	3.7 / 3.7
	114	0.1536	0.0911	0.1041	0.1041	1.2469	9.6 / 6.8
	283	0.1513	0.1073	0.1191	0.1192	1.3193	1.2 / 1.2
	282	0.1596	0.1145	0.1271	0.1272	1.3439	1.1 / 1.1
	280	0.1536	0.0909	0.1038	0.1038	1.2578	5.5 / 4.1
	281	0.1418	0.0929	0.1031	0.1032	1.2642	11.1 / 8.3

[1] population (pop) numbers correspond to voucher numbers in Table 1

[2] H_{obs} = mean observed heterozygosity; H_{exp} = mean expected heterozygosity; H = mean heterozygosity over loci for each population; π = Nucleotide diversity. K = mean number of alleles over loci for each population; A_p = number of private alleles, given as nearly fixed (i.e., present in at least 75% of the individuals) / fixed (i.e., present in all individuals), corrected by nucleotide diversity as described in Westergaard *et al.* (2019).

MOLECULAR ECOLOGY

Table S2 Pairwise F_{ST} values for *Pedicularis asplenifolia* and *Carex fuliginosa* based on SNP data.

Pedicularis asplenifolia

pop[1]	83	107	66	113	286	285
107	0.210					
66	0.166	0.200				
113	0.199	0.254	0.008			
286	0.286	0.332	0.106	0.099		
285	0.278	0.310	0.109	0.094	0.012	
287	0.246	0.301	0.080	0.061	0.116	0.104

Carex fuliginosa

pop[1]	279	82	362	284	71	114	283	282	280
82	0.207								
362	0.314	0.374							
284	0.234	0.231	0.405						
71	0.187	0.204	0.346	0.167					
114	0.213	0.212	0.389	0.205	0.148				
283	0.168	0.200	0.340	0.183	0.152	0.182			
282	0.146	0.206	0.324	0.179	0.134	0.152	0.136		
280	0.192	0.253	0.363	0.211	0.151	0.228	0.168	0.087	
281	0.250	0.268	0.422	0.254	0.197	0.187	0.230	0.109	0.211

[1] population (pop) numbers correspond to voucher numbers in Table 1

MOLECULAR ECOLOGY

Table S3 Comparison of Pleistocene survival scenarios of the study species using two individuals per population and one population less for *Carex fuliginosa*.

Species	Model[1]	Marginal density	Bayes factor[2]	*P*-value
P. asplenifolia	Nun	9.73×10^{-20}	>100	<0.001
	$\text{Peri}_{\text{East}}$+Nun	1.07×10^{-4}	0	0.987
	$\text{Peri}_{\text{South}}$+Nun	3.08×10^{-8}	>100	0.002
	Peri+$\text{Nun}_{\text{Central}}$	2.63×10^{-13}	>100	<0.001
	$\text{Peri}_{\text{East}}$	1.11×10^{-6}	96.39	0.031
	$\text{Peri}_{\text{South}}$	7.48×10^{-6}	14.30	0.065
C. fuliginosa	Nun	4.28×10^{-98}	>100	<0.001
	$\text{Peri}_{\text{East}}$+Nun	2.78×10^{-5}	1.97	0.112
	$\text{Peri}_{\text{South}}$+Nun	5.79×10^{-7}	94.65	0.034
	Peri+$\text{Nun}_{\text{Central}}$	6.85×10^{-115}	>100	<0.001
	$\text{Peri}_{\text{East}}$	5.48×10^{-5}	0	0.486
	$\text{Peri}_{\text{South}}$	7.84×10^{-7}	69.89	0.023

[1] Nun, nunatak survival in interior refugia, the index indicating (where necessary) the location of the ancestral population (Central, central Alps); Peri, peripheral survival in peripheral refugia, the index indicating (where necessary) the location of the ancestral population (East, eastern Alps; South, southern Alps).
[2] the ratio between marginal densities of the best model (i.e., the one with the highest marginal density) and of the alternative model: the higher the value, the higher the support for the best model.

MOLECULAR ECOLOGY

Fig. S1 Plots of (A, C) Delta *K* and of (B, D) mean $Log_e(X|K)$ for each *K* value for (A, B) *P. asplenifolia* and for (C, D) *C. fuliginosa*.

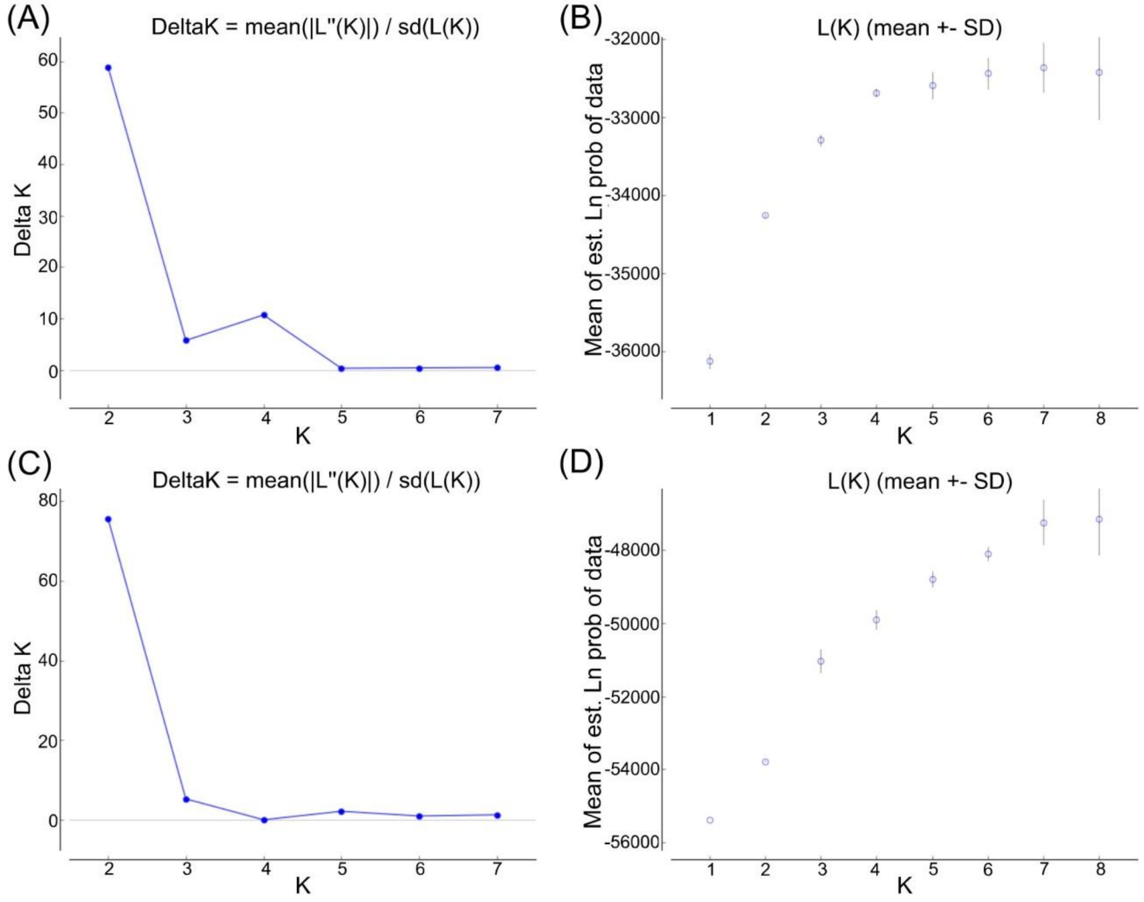

MOLECULAR ECOLOGY

Fig. S2 Genetic structure (K=2) of (A) *Pedicularis asplenifolia* and (B) *Carex fuliginosa* estimated using STRUCTURE. Each column corresponds to one individual. Population numbers correspond to voucher numbers in Table 1.

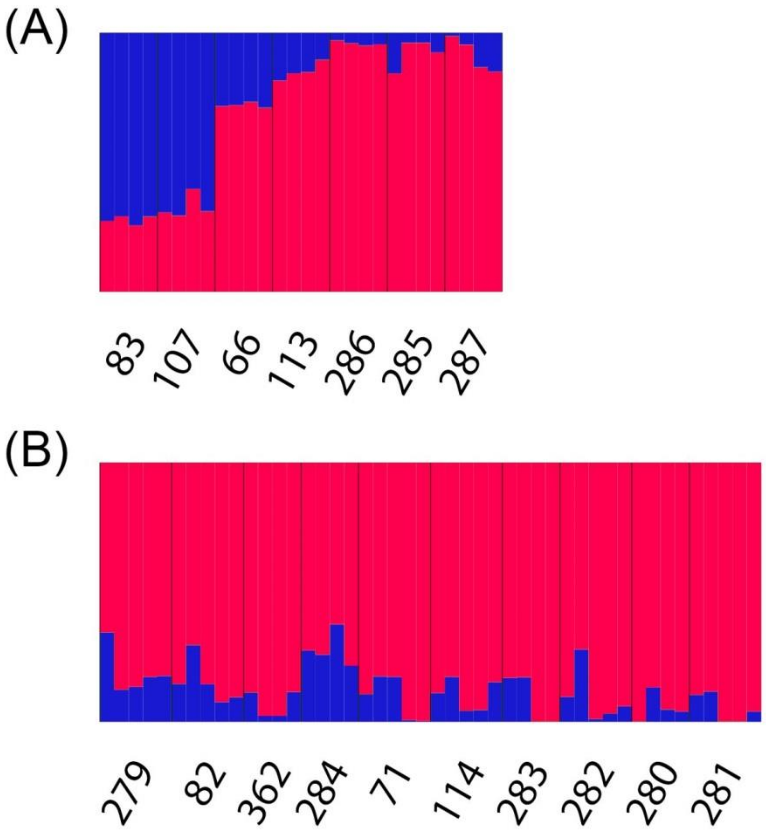

MOLECULAR ECOLOGY

Fig. S3 Neighbor-Net for (A) *P. asplenifolia* and (B) *C. fuliginosa*. Population numbers correspond to voucher numbers in Table 1.

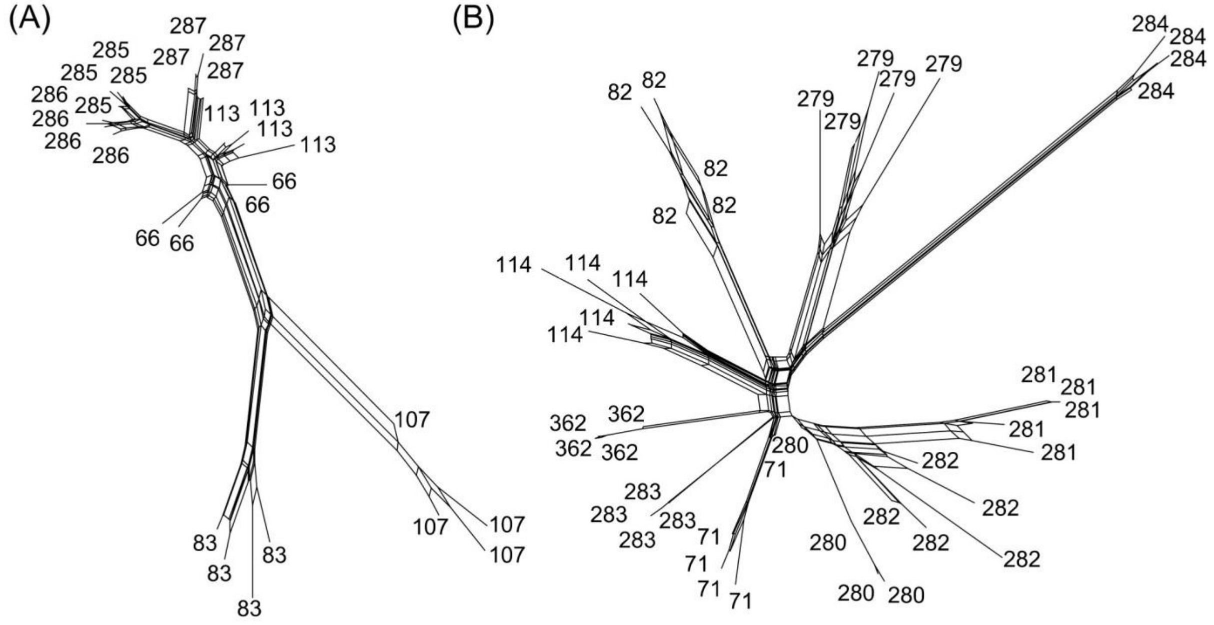

MOLECULAR ECOLOGY

Fig. S4 Scatter-plots of the first two components of a principal component analysis (PCA) for (A) *P. asplenifolia* and (B) *C. fuliginosa* based on SNP data. Population numbers correspond to voucher numbers in Table 1.

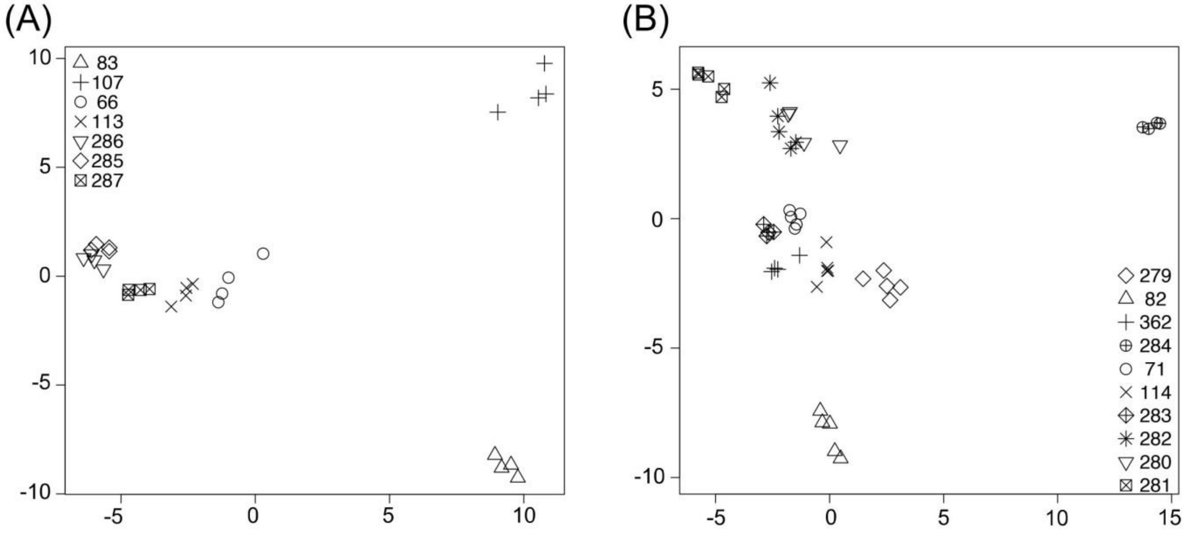

MOLECULAR ECOLOGY

Fig. S5 Change in Root Mean Square Error (RMSE) with increasing number of Partial Least Squares (PLS) components for the most supported model in (A) *Pedicularis asplenifolia* (peripheral plus nunatak survival model with the ancestral population located in the eastern Alps) and (B) *Carex fuliginosa* (peripheral survival only model with the ancestral population located in the eastern Alps). *Nanc* = population size of the ancestral population; *k* = maximum carrying capacity; *m* = migration rate.

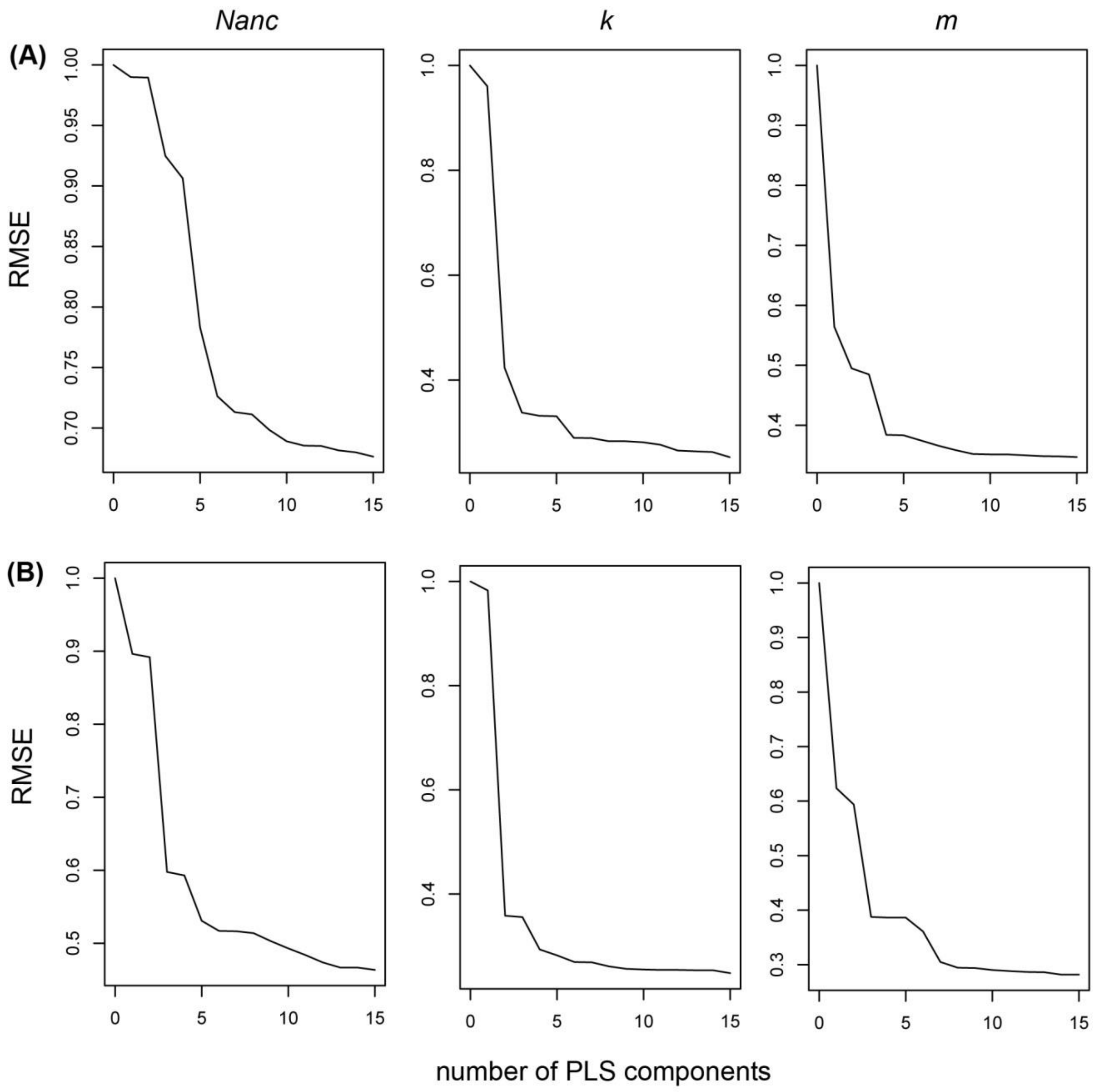

MOLECULAR ECOLOGY

Fig. S6 Prior distributions (black line) and posterior distributions (grey line) of parameter estimates in (A) *Pedicularis asplenifolia* (peripheral plus nunatak survival model with the ancestral population located in the eastern Alps: $\mathrm{Peri}_{\mathrm{East}}$+Nun) and (B) *Carex fuliginosa* (peripheral survival only model with the ancestral population located in the eastern Alps: $\mathrm{Peri}_{\mathrm{East}}$). *Nanc* = population size of the ancestral population; *k* = maximum carrying capacity; *m* = migration rate.

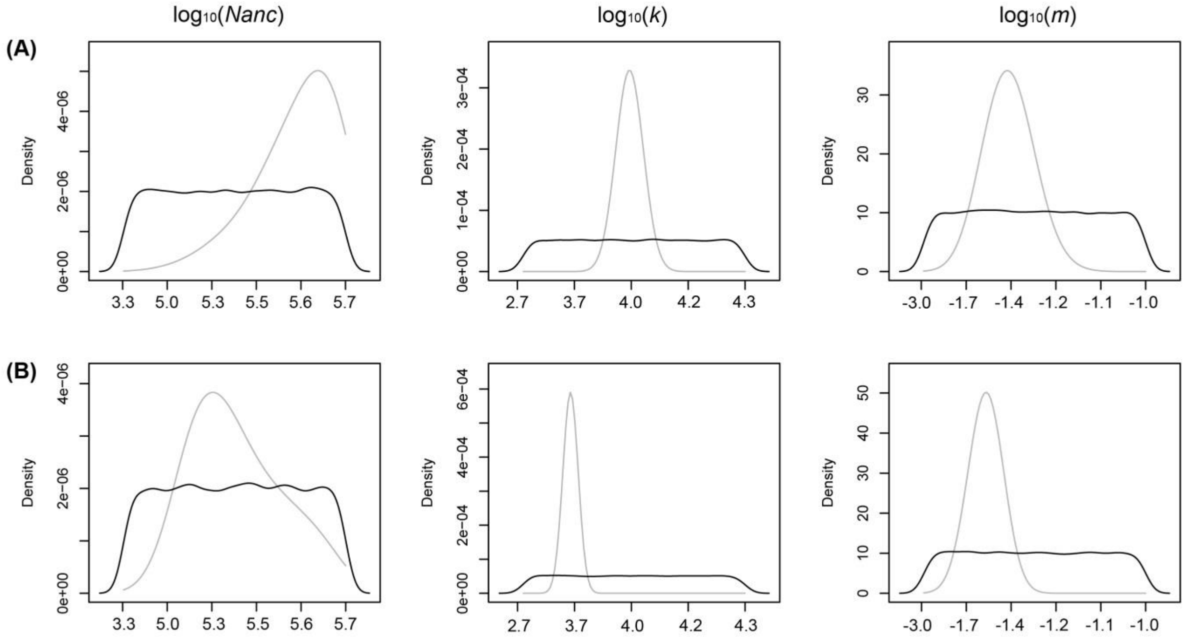

MOLECULAR ECOLOGY

Fig. S7 Distribution of posterior quantiles of parameters for (A) *Pedicularis asplenifolia* (peripheral plus nunatak survival model with the ancestral population located in the eastern Alps: $\text{Peri}_{\text{East}}$+Nun) and (B) *Carex fuliginosa* (peripheral survival only model with the ancestral population located in the eastern Alps: $\text{Peri}_{\text{East}}$). Departures from a uniform distribution were tested with a Kolmogorov-Smirnov test (*p*-values <0.001 in all six tests). *Nanc* = population size of the ancestral population; *k* = maximum carrying capacity; *m* = migration rate.

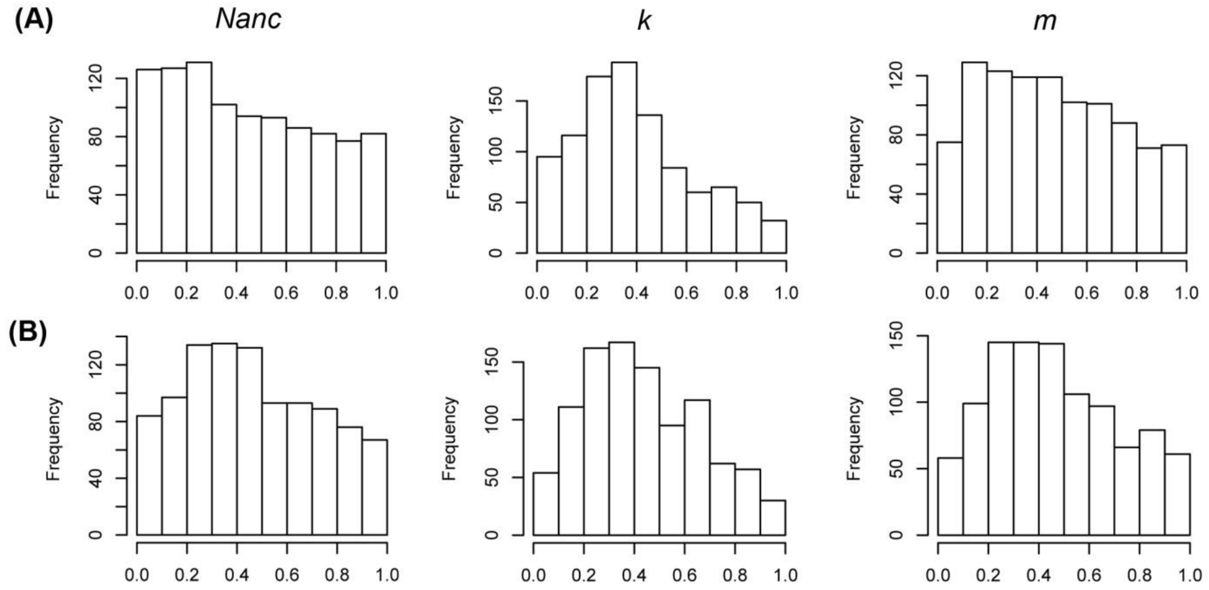

Supplement: Supplementary file 1 [file MEC-29-172-s001.pdf]
